# Individual differences in processing ability to transform visual stimuli during the mental rotation task are closely related to individual motor adaptation ability

**DOI:** 10.3389/fnins.2022.941942

**Published:** 2022-11-14

**Authors:** Tomotaka Ito, Masanori Kamiue, Takayuki Hosokawa, Daisuke Kimura, Akio Tsubahara

**Affiliations:** ^1^Department of Physical Therapy, Faculty of Rehabilitation, Kawasaki University of Medical Welfare, Kurashiki, Japan; ^2^Doctoral Program in Rehabilitation, Graduate School of Health Science and Technology, Kawasaki University of Medical Welfare, Kurashiki, Japan; ^3^Department of Orthoptics, Faculty of Rehabilitation, Kawasaki University of Medical Welfare, Kurashiki, Japan

**Keywords:** mental rotation, laterality judgement, motor imagery, split-belt walking, motor learning, motor adaptation

## Abstract

Mental rotation (MR) is a well-established experimental paradigm for exploring human spatial ability. Although MR tasks are assumed to be involved in several cognitive processes, it remains unclear which cognitive processes are related to the individual ability of motor adaptation. Therefore, we aimed to elucidate the relationship between the response time (RT) of MR using body parts and the adaptive motor learning capability of gait. In the MR task, dorsal hand, palmar plane, dorsal foot, and plantar plane images rotated in 45° increments were utilized to measure the RTs required for judging hand/foot laterality. A split-belt treadmill paradigm was applied, and the number of strides until the value of the asymmetrical ground reaction force reached a steady state was calculated to evaluate the individual motor adaptation ability. No significant relationship was found between the mean RT of the egocentric perspectives (0°, 45°, and 315°) or allocentric perspectives (135°, 180°, and 225°) and adaptive learning ability of gait, irrespective of body parts or image planes. Contrarily, the change rate of RTs obtained by subtracting the RT of the egocentric perspective from that of the allocentric perspective in dorsal hand/foot images that reflect the time to mentally transform a rotated visual stimulus correlated only with adaptive learning ability. Interestingly, the change rate of RTs calculated using the palmar and plantar images, assumed to reflect the three-dimensional transformation process, was not correlated. These findings suggest that individual differences in the processing capability of visual stimuli during the transformation process involved in the pure motor simulation of MR tasks are precisely related to individual motor adaptation ability.

## Introduction

Spatial ability is essential not only for daily activities such as navigation but also for academic achievement such as learning mathematics (Ishikawa and Newcombe, 2021). Mental rotation (MR) is one of the most investigated spatial abilities and is a known cognitive process that is used to mentally manipulate a visual stimulus presented in a rotated state and recognize the shape of a figure (Shepard and Metzler, 1971). In the MR task, several types of pictures/images are used as visual stimuli, such as three-dimensional images (Shepard and Metzler, 1971), alphanumeric characters (Corballis and Sergent, 1989), and body parts (Cooper and Shepard, 1975; Parsons, 1987).

In the field of rehabilitation medicine, the MR task, particularly using body parts, is used as a tool in rehabilitation approaches (Kawasaki and Higuchi, 2013) and assessments (Schwoebel et al., 2001; Johnson et al., 2002; Nico et al., 2004; Fiorio et al., 2006; Coslett et al., 2010; Katschnig et al., 2010; de Vries et al., 2013; Ionta et al., 2016). Intervention using the MR task with foot images has been reported to immediately improve balance ability in healthy people (Kawasaki and Higuchi, 2013). Additionally, rotated hands and feet have been used as MR tasks to objectively assess the motor imagery ability in patients with upper limb amputation (Nico et al., 2004), chronic pain (Schwoebel et al., 2001; Coslett et al., 2010), spinal cord injury (Ionta et al., 2016), stroke (Johnson et al., 2002; de Vries et al., 2013), dystonia (Fiorio et al., 2006; Katschnig et al., 2010), and similar conditions. For instance, upper limb amputees showed slower response times (RTs) than non-amputees in the MR task that required hand laterality judgment (Nico et al., 2004), and patients with leg pain were slower than those with the pain of other body parts in responding to painful foot images (Coslett et al., 2010). These results indicate that the participant's body representation or physical function and ability affect the RTs measured in the MR task, notably the one associated with impaired body parts.

**Figure 1:**
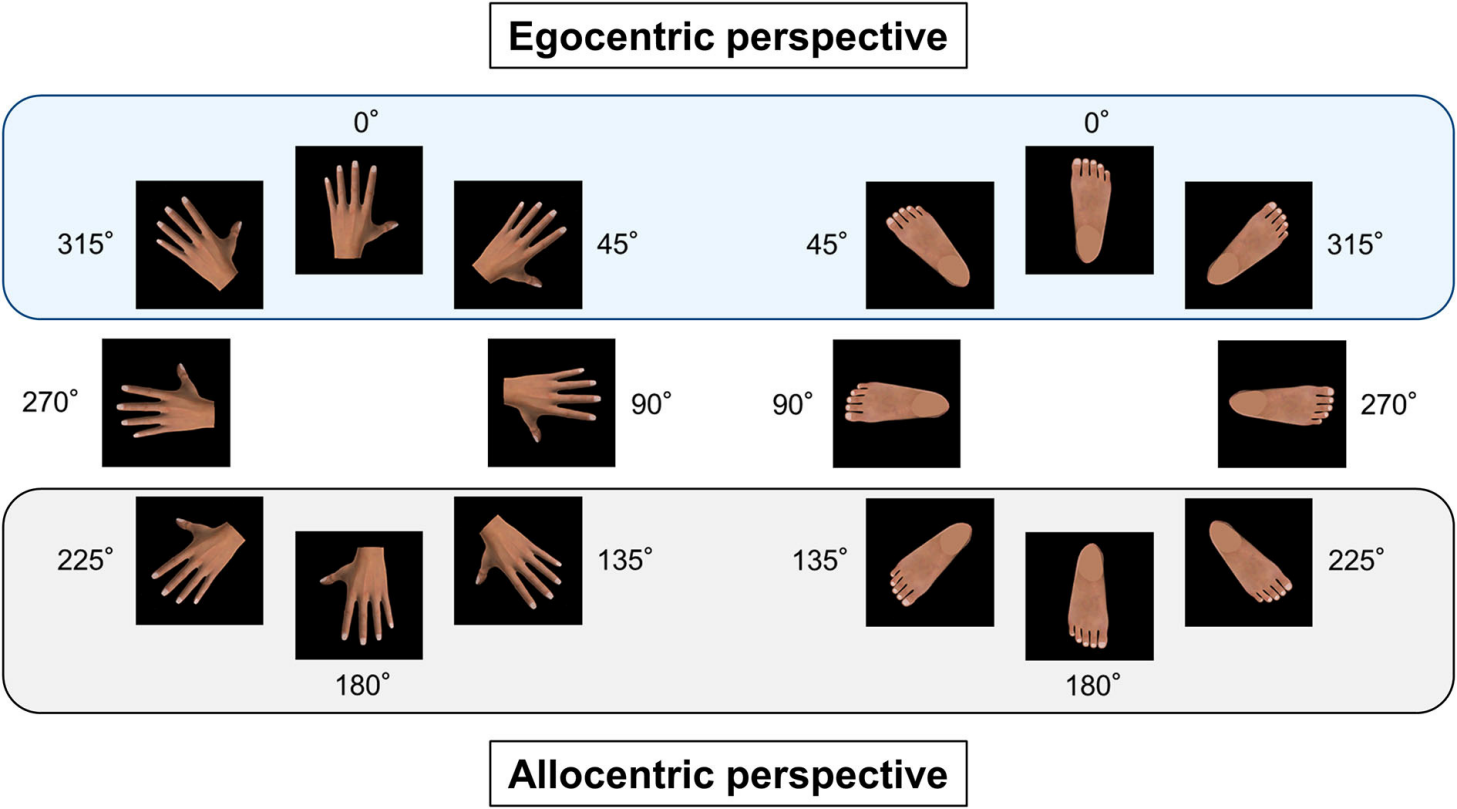
Egocentric and allocentric perspectives of body part images. The left body part images, such as the dorsal hand images, were rotated clockwise, whereas the right body part images, such as the dorsal foot images, were rotated counterclockwise in 45° increments. The visual stimuli were displayed in eight different angular disparities of 0° (upright position), 45°, 90°, 135°, 180°, 225°, 270°, and 315°. The egocentric perspective was comprised of three image orientations: 0°, 45°, and 315°, and the allocentric perspective was comprised of three image orientations: 135°, 180°, and 225°.

The RT of MR tasks is well known to be affected by the depicted images, such as images of body parts (Parsons, 1987) and rotation angles (Sekiyama, 1982; Parsons, 1987). In general, the orientation of body part images around 0° is presented as egocentric (first-person) perspective, whereas those away from 0° and close to 180° are presented as allocentric (third-person) perspective in the MR task (Saxe et al., 2006; Brady et al., 2011; Edwards et al., 2019) (Figure 1). The RT of allocentric images was slower than that of egocentric images, indicating that the RTs in the MR task using body part images were influenced by realistic biomechanical constraints of body movements. Recently, this biomechanical effect was utilized to characterize the alteration of sensorimotor bodily representation in patients with spinal cord injuries (Scandola et al., 2019). Scandola et al. (2019) assessed the changes in biomechanical effect [i.e., the difference in RT between egocentric (0°) and allocentric (180°) perspectives] before and after physiotherapy using foot, hand, and body images and demonstrated that the changes varied with among the pictured body parts. Additionally, individual physical function/ability was reportedly related to the RT of corporal visual stimuli that required large amplitude of rotation (i.e., allocentric images) (Schwoebel et al., 2001; Coslett et al., 2010). Thus, both body parts and rotation angles of the presented images in the MR task appear to be crucial during the assessment of physical function/ability.

Motor learning ability is also a fundamental capability to accomplish diverse daily activities in challenging environments/situations. A growing body of evidence indicates the benefit of mental practice with motor imagery on the performance of motor learning tasks with upper (Gentili et al., 2006; Michel et al., 2013; Kraeutner et al., 2016; Ruffino et al., 2021) or lower limbs (Taube et al., 2014). Furthermore, a questionnaire assessment showed that individuals with higher motor imagery ability were able to acquire new movements with fewer trials than individuals with lower motor imagery ability (Goss et al., 1986). A more direct finding comes from a neurophysiological study using monkeys, which indicates that covert rehearsal/learning, without actual movements, transfers to overt performance, and that covert and overt movements share a common neural substrate (Vyas et al., 2018). These findings appear to emphasize the association between motor imagery and motor learning abilities. Given that the MR task is an implicit method to assess motor imagery ability (Dahm, 2020), motor imagery ability assessed by the MR task and motor learning ability is speculated to be related.

As to the relationship between MR and motor adaptation abilities, the RTs in the MR tasks using a letter (Pellizzer and Georgopoulos, 1993) or cards (Anguera et al., 2010) were found to be significantly correlated with the speed of adaptive performance in a sensorimotor adaptation task that generally requires participants to correct and adapt upper limb movements against a visual perturbation. In the visuomotor adaptation task using the upper extremity, cognitive ability, such as spatial working memory ability, has been found to relate to the capability of adaptive learning (Anguera et al., 2010; Seidler et al., 2012; Christou et al., 2016), and both the visuospatial working memory task involving MR and the visuomotor adaptation task recruit similar neural circuits (Anguera et al., 2010). Although a growing body of evidence has examined the association between MR or cognitive abilities and motor adaptation ability in the upper extremity task, few studies have examined its relevance using a lower extremity task (French et al., 2021), and the relevance is not well known.

Herein, to evaluate the motor adaptation ability, we applied a well-established experimental paradigm using a split-belt treadmill (Reisman et al., 2005; Morton and Bastian, 2006; Choi and Bastian, 2007; Malone and Bastian, 2010; Vasudevan et al., 2011; Bruijn et al., 2012). The split-belt treadmill consists of two different belts that independently move at different velocities. When participants are exposed to a novel walking environment with different belt speeds, due to gradual adaption to the new condition, the adaptive alteration process of dynamic gait motion can be assessed. We selected this split-belt treadmill paradigm as an adaptation learning task because there are apparent individual differences in adaptive motor learning ability even in healthy individuals of the same age.

A recent meta-analysis by Tomasino and Gremese (2015) focused on the stimulus type-specific modulation of brain networks during MR tasks and reported that bodily visual stimuli activated a bilateral sensorimotor network more than non-bodily stimuli. In addition, hand laterality judgment (MR task) has been reported to show comparable brain activities with motor imagery of hand movements (Hamada et al., 2018). These findings support the speculation, discovered in behavioral studies, that when rotated visual stimuli are associated with body parts, participants can mentally rotate their own hands or feet to judge the laterality of displayed body part images in the absence of actual body movements (Cooper and Shepard, 1975; Parsons, 1987). However, MR tasks are presumed to comprise several sequential cognitive processes (Corballis, 1988; Seurinck et al., 2004; Xue et al., 2017), such as (1) visual encoding, (2) transformation, (3) comparison, (4) decision making, and (5) motor response generation (Seurinck et al., 2004). Hence, if RT values that include all these processes were used as an outcome of some assessment, what the obtained results intrinsically reflect and what the MR task actually evaluates would be unknown.

To solve the latent feature that the MR task contains several cognitive processes, first, we configured various factors of the presented image, such as body parts (hand and foot), image plane (dorsal and ventral sides), and image view (egocentric and allocentric perspectives), and especially focused on the image view. Because visual experience: familiarity with or habitual visual exposure to a particular body part, affects the RT of egocentric/allocentric perspective images of hand/foot (Edwards et al., 2019), we assumed that the RT of different perspective and body part images reflects individual variations in processing ability to encode or perceive the corporal visual stimuli (i.e., visual encoding process). Second, we handled the obtained RT data to disclose the effect of a particular cognitive process by attempting to discriminate the process of the MR task as much as possible. The aforementioned biomechanical effect (i.e., the difference in RT between egocentric and allocentric perspectives) of the MR task was used as a hallmark of motor involvement (Conson et al., 2010; Scandola et al., 2019) and is thought to reflect the very mental transformation of body parts itself; therefore, we hypothesized that the transformation process represented by biomechanical effect was intimately connected with individual adaptive learning ability irrespective of the depicted body parts. Moreover, because it has been suggested that ventral side images are more effective than dorsal side images in triggering motor information to mentally transform body parts (Parsons, 1987; Krüger and Krist, 2009), we speculated that the relationship between biomechanical effect and learning ability would be more pronounced in the ventral side images than the dorsal side images.

Taken together with the finding that common brain regions, such as the motor-related area and parietal association area, are activated during both the MR task of body parts (de Lange et al., 2006; Creem-Regehr et al., 2007; Corradi-Dell'Acqua et al., 2009; Perruchoud et al., 2016; Hamada et al., 2018; Qu et al., 2018) and the split-belt treadmill walking task (Hinton et al., 2019), the latent relationship between these two tasks was likely to be strong. Thus, this study aimed to advance our understanding and elucidate the elements or processes of the MR task that were related to individual ability of motor adaptation on gait. Studies in this direction would be helpful when we apply the MR task of body parts to improve physical ability or to predict an individual's adaptive motor learning ability prior to the actual intervention.

## Experimental procedures

### Participants

Thirty-one young healthy adults (18 women, mean age ± SD: 20.6 ± 0.6 years) without any history of orthopedic or neurological diseases and naive to MR task and split-belt treadmill walking were included. The dominant hand of each participant was assessed using the Edinburgh Handedness Inventory (Oldfield, 1971) (right hand dominant: 30 participants). This study conformed to the principles of the Declaration of Helsinki and was approved by the ethics committee of the Kawasaki University of Medical Welfare (approval number: 18-039). All participants provided written informed consent.

### Measurements

The time required to walk 10 m was measured two times using a stopwatch. The slower time of the two measurements was used to set the belt speed of a split-belt treadmill (Bertec Co., Columbus, OH, USA). After measuring the participants' comfortable walking speed, an MR task that uses arrows and body parts was performed, and the gait adaptation process was measured using a split-belt treadmill to evaluate the learning ability of each participant. The data of the MR task and learning ability were collected on the same day.

### MR task

During the MR task, participants sat on a chair with a backrest facing a 15.6-inch monitor of a personal computer (L560; Lenovo Japan Ltd., Tokyo, Japan), which presented visual stimuli, with their hands placed on each button to respond to the laterality, and was hidden from view by a box. The participants were instructed to press the right button with their right hand as quickly and accurately as possible when the right images of visual stimuli were displayed. Similarly, when the left images of the visual stimuli were presented, they were instructed to use their left hands to press the left button. They were also instructed not to move their bodies when judging the laterality of the images.

Left and right images of the dorsal hand, palmar plane, dorsal foot, plantar plane, and arrow were used as visual stimuli to judge laterality. The left hand and foot images were rotated clockwise, whereas the right hand and foot images were rotated counterclockwise in 45° increments. Hence, each hand and foot image comprised eight different rotation angles: 0° (upright position), 45°, 90°, 135°, 180°, 225°, 270°, and 315° (Figure 1). Individual hand and foot images were repeatedly presented three times in a pseudorandom order, and the left and right directions of the arrow images were displayed on the monitor 10 times each. Therefore, 96, 96, and 20 visual stimuli were used for the laterality judgment task of the hands, feet, and arrows, respectively. We recorded both the RT, defined as the time between the display of the stimulus image and the participant pressing the right or left button using their hand, and the error rate as an index of accuracy. Randomized presentation of visual images, measurement of RTs, and storage of true–false results were performed using a customized stimulus presentation software (Takei Scientific Instruments Co., Ltd., Niigata, Japan). Prior to the MR task, all participants practiced judging whether a presented image was left or right using a set of 20 images. To avoid habituation of the judging body part laterality, we prepared separate images for practice, including four different rotation angles (60°, 120°, 240°, and 300°) of the hand and foot images and the arrow images. The MR tasks of the hand, foot, and arrow images were conducted individually, and the order of each MR task was random. Considering that fatigue might affect the participants' concentration, a 2-min rest time was provided between each MR task. The measured RTs of each image were preserved on a personal computer and used for offline analysis.

### Split-belt walking

**Figure 2:**
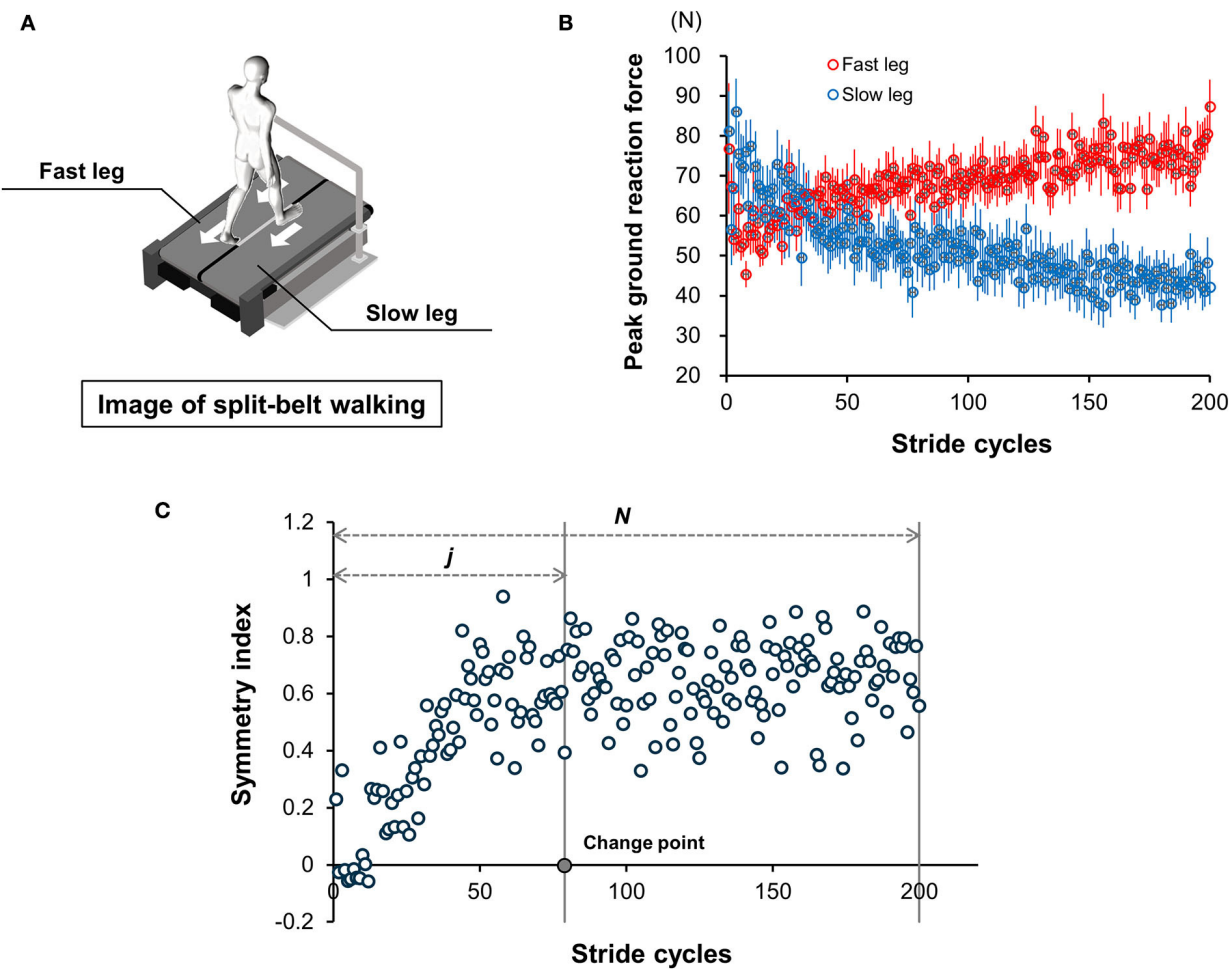
Split-belt walking task. **(A)** Graphical representation of the split-belt walking task under different belt speed conditions. Fast leg was defined as the leg on the fast-speed belt, and slow leg was defined as the leg on the slow-speed belt in this study. **(B)** Mean peak values of posterior (Fy) component of the ground reaction force during each stance phase as an illustration (*N* = 15). Red circles indicate the changes in mean peak values with the fast leg. Blue circles are those with slow leg. Error bars display standard errors. **(C)** Representative change in SI during split-belt treadmill walking and change point obtained from a single participant. Using the mean peak values obtained from the bilateral leg sides, the symmetry index (SI) was calculated to examine the individual gait adaptation process, as follows: SI = (fast leg – slow leg)/(fast leg + slow leg).

The participants were randomly assigned to the two walking conditions. The ratio of the right to left belt speed (right: left) was set at 3:1 (right fast condition) or 1:3 (left fast condition) based on the aforementioned preferred walking speed. The participants wore a safety harness and were oriented with one leg on each belt. The leg on the fast-speed belt was defined as the “fast leg” and that on the slow-speed belt was defined as the “slow leg” (Figure 2A). Before an asymmetrical walk on the treadmill, the participants practiced walking on it at their preferred walking speed for 1 min and subsequently walked on it for 5 min under two different belt speeds. Data of three-dimensional ground reaction force (GRF) components, mediolateral (Fx), anteroposterior (Fy), and vertical (Fz), were separately recorded from the right and left sides of the force plates built on the treadmill at a frequency of 1,000 Hz.

### Data analysis

**Figure 3:**
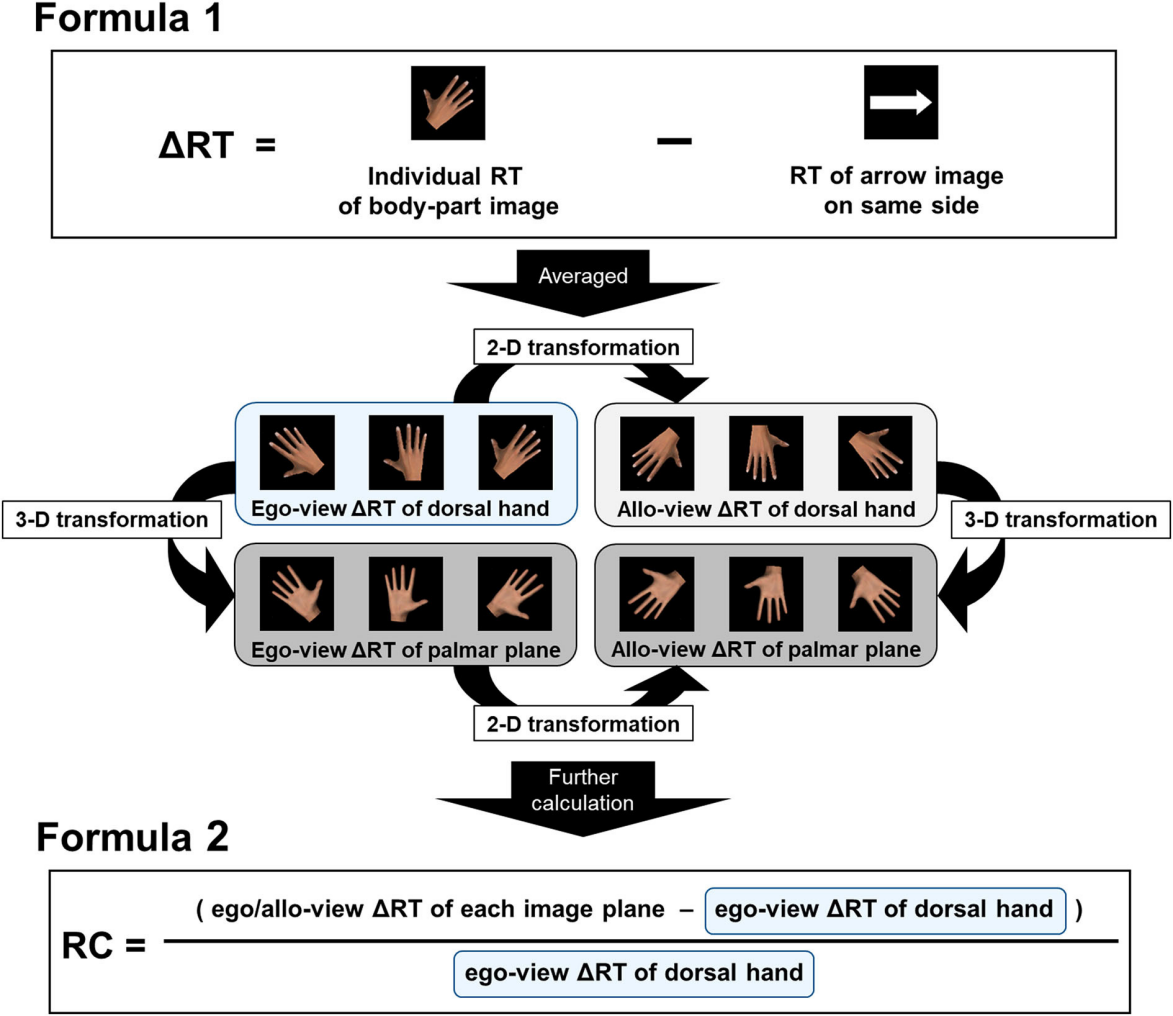
Illustration of data handling. Herein, right-hand image is shown as an example. RT of the arrow image (arrow RT) on the same side was subtracted from individual raw RT data for the body part images (ΔRT; Formula 1). Averaged RTs for egocentric views (ego-view ΔRT; 0°, 45°, and 315°) and allocentric views (allo-view ΔRT; 135°, 180°, and 225°) were then individually calculated. As shown in the center of the figure, the two-dimensional (2-D) or three-dimensional (3-D) transformation process in the MR task was assumed in reference to the ego-view ΔRT of the dorsal side body part images (reference value). The rate of change (RC) was calculated by subtracting the reference value from the ego/allo-view ΔRT of each image plane and dividing the value by the reference one (Formula 2) and used as an indicator of the transformation process.

One participant showed an error rate of over 20%, equal to three standard deviations away from the mean, in the foot image. Therefore, the data obtained from this participant were excluded, and data from 30 participants were used in the analyses. The RTs of each plane and rotation angles for the hand and foot images were averaged by the correct response values from three presentations (raw RT data). The RTs of the right and left arrow images were also calculated by averaging the latter half of the values from 10 presentations (arrow RT), and individual raw RT data for the hand and foot were subtracted by the arrow RT on the same side (ΔRT) (Figure 3). Since Edwards et al. (2019) demonstrated that individual features, such as visual or motor experience, differentially influence the RT of egocentric/allocentric perspective images, we focused on this intriguing point to characterize the individual variations in RTs. Therefore, after prior calculations, we further averaged the ΔRTs for egocentric views (ego-view ΔRT; 0°, 45°, and 315°) and allocentric views (allo-view ΔRT; 135°, 180°, and 225°) individually (Figure 3). Based on the data handling by Conson et al. (2013), we regarded the ego-view ΔRT of dorsal hands and feet as a time window reflecting the pure visual encoding or perception process of the body part image. Additionally, to highlight the process of mental spatial transformation of the hands and feet and exclude the effect of the visual encoding process from each ΔRT value, we calculated the rate of change (RC), using dorsal hand and foot images, by subtracting ego-view ΔRT from allo-view ΔRT and dividing the result by ego-view ΔRT for statistical analyses (hereafter mentioned as RC of hand ΔRT and RC of foot ΔRT, respectively) (Figure 3). Similarly, to examine more complex biomechanical effects (i.e., three-dimensional transformation process), the RCs with palmar and plantar plane images were also calculated using the following formula (hereafter mentioned as RC of palm-ego ΔRT, RC of palm-allo ΔRT, RC of planta-ego ΔRT, and RC of planta-allo ΔRT, respectively) (Figure 3):

$$\begin{array}{l} \text{RC of palm-allo }\Delta \text{RT}\\ =\text{(allo-view }\Delta \text{RT of palmar plane}-\text{ego-view }\Delta \text{RT of dorsal}\\ \text{ hand)}/\text{ego-view }\Delta \text{RT of dorsal hand}. \end{array}$$

Further, to identify the stance phase during each stride cycle, the timing of heel contact and toe-off was detected from Fz of GRF (threshold: 20 N), according to Yokoyama et al. (2018). The stepping movement from a static standing position, generated by the commencement of the treadmill, was excluded from the count of steps for data analyses. In this study, the first step was defined as the ground contact of the contralateral leg following the stepping movement, and this first step and the subsequent second step were together regarded as the initial stride cycle. After the identification of the stride cycle, the peak value of the backward braking force was extracted from Fy of the GRF during the individual stance phases of fast and slow legs (Ogawa et al., 2014; Yokoyama et al., 2018) (Figure 2B). Using this peak value obtained from both legs, we calculated the symmetry index (SI) values in GRF to examine the alteration of stride symmetry and clarify the individual gait adaptation process, as follows (Yokoyama et al., 2018):

$$\begin{array}{l} \text{SI}=\left(\text{fast leg }-\text{ slow leg}\right)/\left(\text{fast leg }+\text{ slow leg}\right) \end{array}$$

Subsequently, using the SI values, the change point (CP) of each participant was calculated to obtain the number of stride cycles required for adaptation (Figure 2C) and evaluate the participant's learning ability on gait under unusual walking conditions. The CP was defined as the point at which the changes in SI reached a steady state after an increase in SI values, obtained using the following formula (Siegel and Castellan, 1988):

$$\begin{array}{l} \text{CP }=\max|2\text{Wj }-\text{ j}\left(\text{N }+\;1\right)| \end{array}$$

where *j* represents the number of stride cycles, *Wj* represents the cumulative sum of ranks up to the sequence number of stride cycles *j*, and N represents the total number of stride cycles.

The point *j* at which the value was maximum was considered as the CP (Figure 2C). In this study, considering the between-participant difference in the number of stride cycles performed during 5 min of treadmill walking, the N value of all participants was set at 200 (Figure 2C). For evaluating the participants' learning ability, the CP value was divided by N, and the individual learning ability was expressed as a percentage.

### Sample size

The sample size was determined using the G^*^Power software package (version 3.1.9.4, Germany). To elucidate the correlation between the RT of MR and the actual learning ability, the sample size was estimated for an expected effect size of r = 0.50, with an alpha of 0.05, and a power of 0.80 prior to participant recruitment. Because there were some converging evidences substantiating the relationship between the RT of MR and the actual physical function/ability (Schwoebel et al., 2001; Johnson et al., 2002; Nico et al., 2004; Fiorio et al., 2006; Coslett et al., 2010; Katschnig et al., 2010; de Vries et al., 2013; Ionta et al., 2016), we selected the large effect size to avoid wasting sample resources and beta error probability. Hence, the desired number of participants necessary for this experiment was 29. More than 30 volunteers were recruited due to the probability of data unavailability because of numerous errors in hand/foot laterality judgment. After the completion of the study, a *post-hoc* power analysis was also conducted to confirm the validity of the estimated sample size; the analysis indicated that 30 participants are suitable to detect the correlation results with a desired statistical power >0.80.

### Statistical analysis

Statistical analyses were performed using SPSS Statistics software, version 22 (IBM Co.: Armonk, NY, USA). We used the Shapiro–Wilk normality test to check whether the data of RT and learning ability were normally distributed. According to the results of the normality test, parametric or non-parametric tests were used for the following statistical analyses. First, to confirm the reliability of the MR task data, the Spearman's rank correlation between the RTs and error rates in the hand and foot images was conducted to rule out a possible speed-accuracy tradeoff (Conson et al., 2014): the faster the MR task is to be completed, the less precisely it tends to be done, and vice versa (Kail, 1985; Hertzog et al., 1993). Second, to demonstrate that the use of raw RT data would distort the results due to individual differences in response speed to stimuli itself, the Spearman's rank coefficient was used to examine the correlation between the raw RTs of egocentric view in the dorsal hand/foot image and the arrow image, respectively. Following this, to show that the ΔRT used in this study (i.e., the difference in RT between the body part and arrow images) would reflect the time window derived from visual encoding or perception of pure body part image, the Friedman test and *post-hoc* analysis using the Wilcoxon signed-rank test with Bonferroni correction were performed to disclose the discrepancy between the raw RTs of the egocentric view in the body part image and RTs of the arrow image. Then, to examine the effects of perspectives in different body image planes on ΔRT, the Friedman test was used to compare the ego-view and allo-view ΔRTs in each body image plane (eight conditions). The Wilcoxon signed-rank test with Bonferroni correction was then conducted for multiple comparisons. As the main analysis, to explore the relationship between the various RTs and learning ability on gait, Pearson's correlation coefficient was applied when the data followed a normal distribution; otherwise, its non-parametric alternative, Spearman's rank coefficient, was applied. The level of statistical significance for all analyses was set at α = 0.05.

## Results

### RT in each image plane and perspective

**Figure 4:**
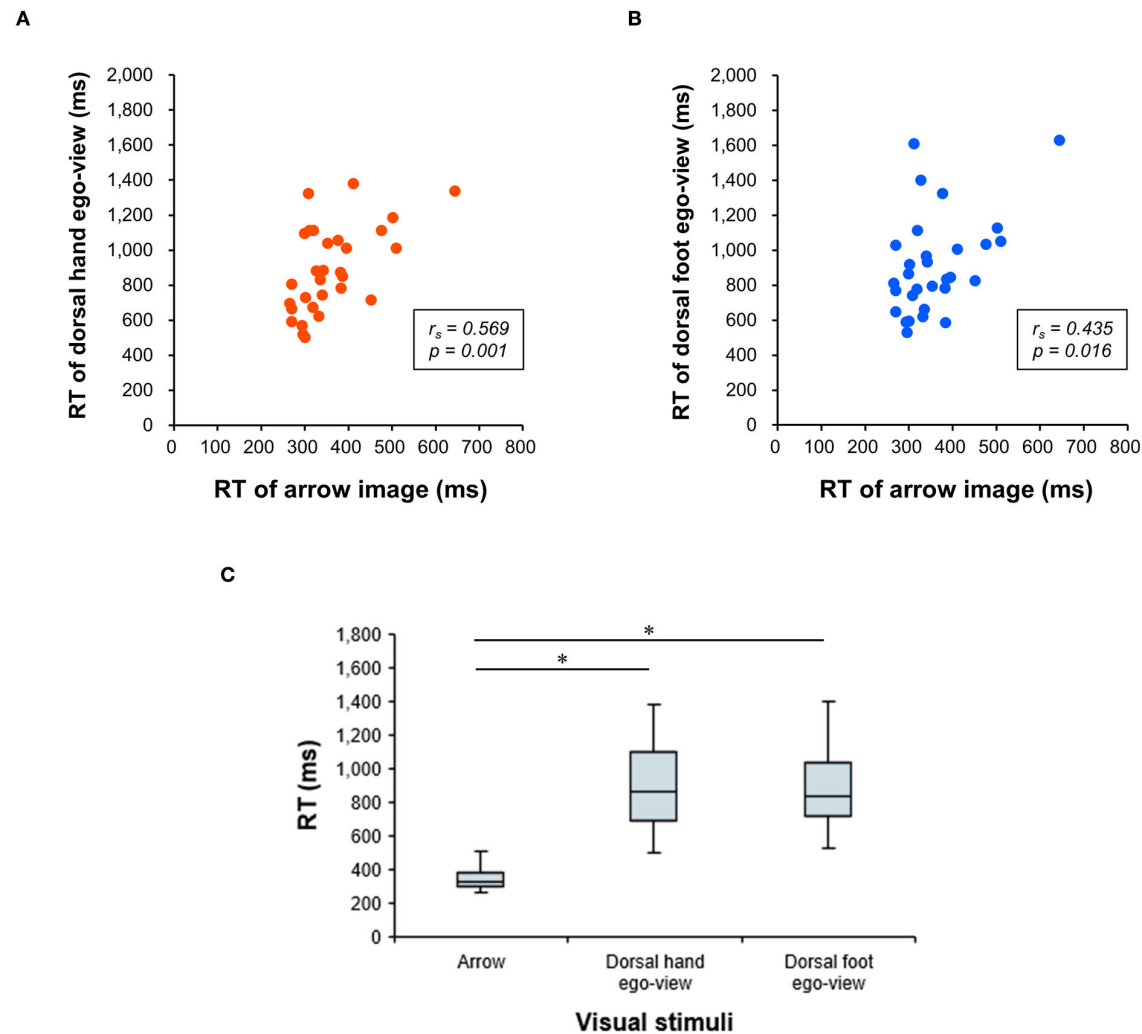
Relationship between RTs of the arrow and dorsal hand/foot images in the egocentric view. Results of the correlation analysis are depicted in the upper row. The x-axis denotes the response time (RT) for the arrow image. The y-axis denotes the RT for the egocentric perspective (ego-view) in the dorsal hand image **(A)** or that for the ego-view in the dorsal foot image **(B)**, respectively. **(C)** Results of comparisons among the RTs of three visual stimuli (arrow, hand, and foot images) are depicted on the lower row. The x-axis denotes a condition of the visual stimuli. The y-axis denotes the RT. * Significant differences with *p* < 0.01.

The median error rates were 2.1% (interquartile range, 1.0–6.0) in the hand image and 4.2% (interquartile range, 3.1–8.3) in the foot image. Correlation analyses revealed significant positive correlations between the RTs and error rates in both the hand (r_s_ = 0.396, *p* = 0.030) and foot (r_s_ = 0.432, *p* = 0.017) images. These results cannot be attributed to a speed-accuracy tradeoff. Significant positive correlations were also found between the RT of the arrow and the raw RT data of the egocentric view, both in the dorsal hand (r_s_ = 0.569, *p* = 0.001; Figure 4A) and the foot (r_s_ = 0.435, *p* = 0.016; Figure 4B) images. In addition, the RTs of the egocentric view in the dorsal hand and the foot images were shown to be significantly slower than the RT of the arrow image (all *p* < 0.001, r = 0.873; Figure 4C). Taking these results together, the RT of the arrow image unrelated to the body part may be suitable stimuli to eliminate individual differences in the generation of the motor response (i.e., simple stimulus-response), and ΔRT in this study (i.e., the difference in RT between body part and arrow images) would be a rational indicator reflecting the visual encoding or the body part perception process.

**Figure 5:**
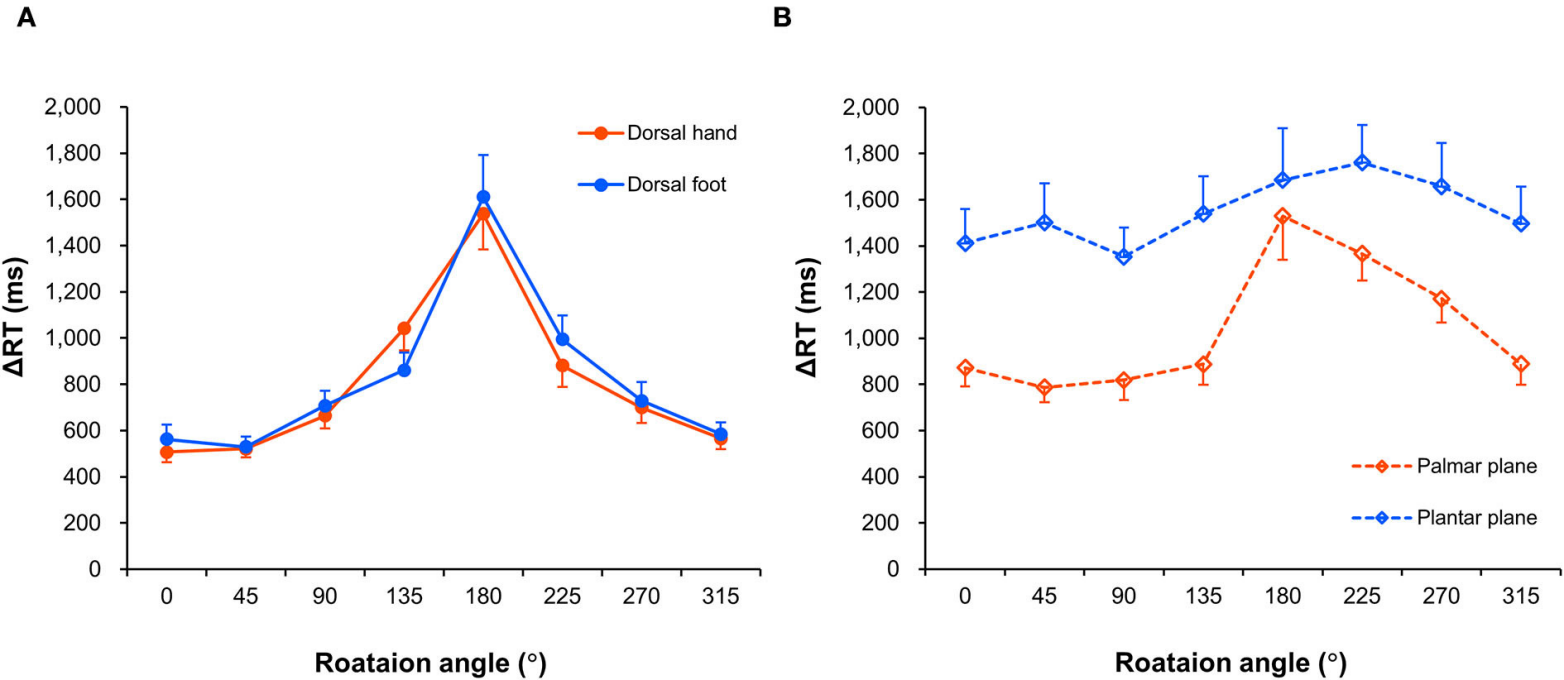
Mean response times. Change in response times (ΔRTs) of the dorsal hand and foot images **(A)** and the palmar plane and plantar plane images **(B)** across each rotation angle. Mean ΔRTs were calculated by subtracting mean RT values for the arrow images from the mean RT values for each body part image. The data are shown as mean and standard error.

**Figure 6:**
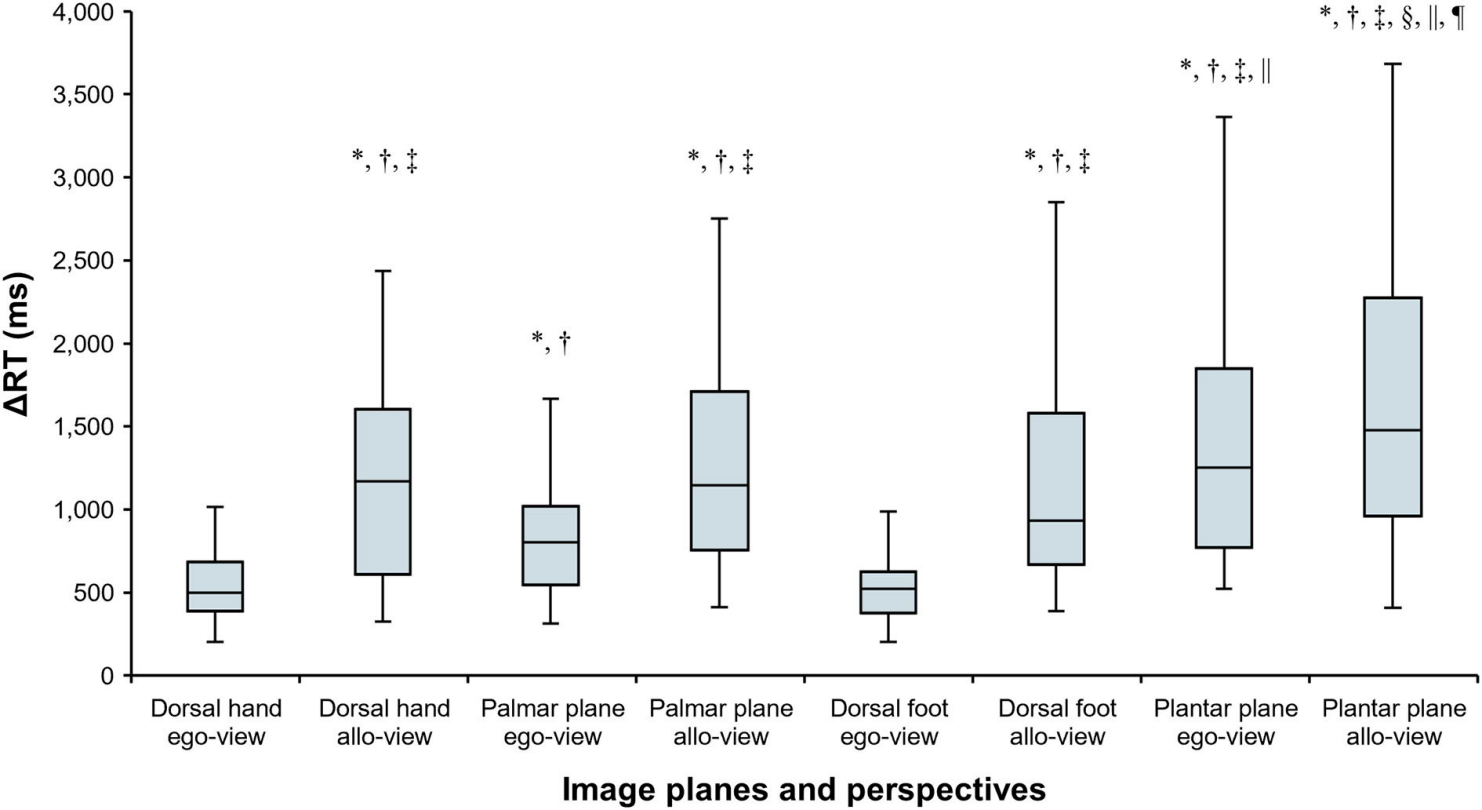
Comparisons of ΔRTs in individual image planes (dorsal hand, palmar plane, dorsal foot, and plantar plane) and perspectives (ego-view and allo-view). Data are shown as median and interquartile range. * response time (ΔRT) is significantly slower than that in the egocentric view (ego-view) of the dorsal hand. ^†^ ΔRT is significantly slower than that in the ego-view of the dorsal foot. ^‡^ ΔRT is significantly slower than that in the ego-view of the palmar plane. ^§^ ΔRT is significantly slower than that in the allocentric view (allo-view) of the dorsal hand. ^||^ ΔRT is significantly slower than that in the allo-view of the dorsal foot. ^¶^ ΔRT is significantly slower than that in the allo-view of the palmar plane (all *p* < 0.002).

Figure 5 shows the RTs for eight different rotation angles of the four body image planes, indicating that the alteration of RTs generated by angular disparity depends on the displayed body image planes. The Friedman test revealed that there was a statistically significant difference among ΔRTs in individual image planes and perspectives (χ^2^_(7)_ = 158.200, *p* < 0.001, Kendall's *W* = 0.753). The results of multiple comparison with the Wilcoxon signed-rank test are shown in Figure 6, and *p* values and effect sizes (r) are shown in Table 1. First, regarding the ΔRTs of the dorsal hand and foot image planes, the allo-view ΔRT was significantly slower than ego-view ΔRT (all *p* < 0.001). However, no significant difference was found between the ΔRTs of the dorsal hand and foot image planes irrespective of perspectives. These results denote that the RTs of the dorsal body part images were considerably similar. Second, in the ΔRT of the palmar plane images, the allo-view ΔRT was significantly slower than the ego-view ΔRT of the same image plane (*p* < 0.001), as well as the ego-view ΔRTs of the dorsal hand and foot images (all *p* < 0.001). A statistically significant difference was also detected between the ego-view ΔRTs of the palmar plane and dorsal body part images (all *p* ≤ 0.001), indicating that the difference in ΔRT is prone to be large in the egocentric views relative to the allocentric views. Last, in the ΔRT of the plantar plane images, the allo-view and ego-view ΔRTs of plantar plane images were significantly slower than the ego-view ΔRTs of the dorsal hand, palmar plane, dorsal foot images, and the allo-view ΔRT of dorsal foot image (all *p* ≤ 0.001). Additionally, the allo-view ΔRT of plantar plane images was significantly slower than the allo-view ΔRTs of the dorsal hand and palmar plane images (all *p* < 0.001). These findings demonstrate that the RTs of the plantar plane images are evidently slow compared to the other body-plane images.

**Table 1:** Results of multiple comparisons between ΔRTs in eight different body part images.

**Comparison**		***P***	**Effect size (r)**
Dorsal hand ego-view ΔRT	vs. Dorsal hand allo-view ΔRT	**<0.001**	0.873
	vs. Dorsal foot ego-view ΔRT	0.428	0.145
	vs. Dorsal foot allo-view ΔRT	**<0.001**	0.847
	vs. Palmar plane ego-view ΔRT	**<0.001**	0.832
	vs. Palmar plane allo-view ΔRT	**<0.001**	0.873
	vs. Plantar plane ego-view ΔRT	**<0.001**	0.873
	vs. Plantar plane allo-view ΔRT	**<0.001**	0.873
Dorsal foot ego-view ΔRT	vs. Dorsal hand allo-view ΔRT	**<0.001**	0.873
	vs. Dorsal foot allo-view ΔRT	**<0.001**	0.873
	vs. Palmar plane ego-view ΔRT	**<0.001**	0.787
	vs. Palmar plane allo-view ΔRT	**<0.001**	0.873
	vs. Plantar plane ego-view ΔRT	**<0.001**	0.873
	vs. Plantar plane allo-view ΔRT	**<0.001**	0.873
Palmar plane ego-view ΔRT	vs. Dorsal hand allo-view ΔRT	**<0.001**	0.712
	vs. Dorsal foot allo-view ΔRT	**0.001**	0.629
	vs. Palmar plane allo-view ΔRT	**<0.001**	0.734
	vs. Plantar plane ego-view ΔRT	**<0.001**	0.858
	vs. Plantar plane allo-view ΔRT	**<0.001**	0.869
Plantar plane ego-view ΔRT	vs. Dorsal hand allo-view ΔRT	0.021	0.422
	vs. Dorsal foot allo-view ΔRT	**0.001**	0.584
	vs. Palmar plane allo-view ΔRT	0.116	0.287
	vs. Plantar plane allo-view ΔRT	0.049	0.359
Dorsal hand allo-view ΔRT	vs. Dorsal foot allo-view ΔRT	0.877	0.028
	vs. Palmar plane allo-view ΔRT	0.185	0.242
	vs. Plantar plane allo-view ΔRT	**<0.001**	0.708
Dorsal foot allo-view ΔRT	vs. Palmar plane allo-view ΔRT	0.271	0.201
	vs. Plantar plane allo-view ΔRT	**<0.001**	0.835
Palmar plane allo-view ΔRT	vs. Plantar plane allo-view ΔRT	**<0.001**	0.640

Ego, egocentric; allo, allocentric; RT, response time.

*P* value was adjusted to 0.002 with the Bonferroni correction. The bold values indicate the statistically significant difference values.

### Relationship between learning ability on gait and RT

In the current study, the number of stride cycles necessary for adapting a novel walking condition created by a split-belt treadmill to reach a steady state was calculated to evaluate participants' adaptive motor learning ability and expressed as CP (Figure 2C). First, during correlation analysis examining the relationship between the adaptive learning ability on gait and the primary source data of ΔRT, the ego-view ΔRTs and allo-view ΔRTs in the dorsal hand, palmar plane, dorsal foot, and plantar plane, showed no statistically significant correlations (Table 2).

**Table 2:** Correlation between adaptive learning ability on gait and values of RT.

	**Correlation coefficient**	***P***
**Dorsal hand**		
Ego-view ΔRT	0.030	0.877
Allo-view ΔRT	0.320	0.084
**Palmar plane**		
Ego-view ΔRT	0.263	0.160
Allo-view ΔRT	0.148	0.436
**Dorsal foot**		
Ego-view ΔRT	0.088	0.643
Allo-view ΔRT	0.280	0.134
**Plantar plane**		
Ego-view ΔRT	0.344	0.062
Allo-view ΔRT	0.194	0.305
**RC**		
Hand ΔRT	0.521	**0.003**
Foot ΔRT	0.548	**0.002**
Palm-ego ΔRT	0.269	0.151
Palm-allo ΔRT	0.222	0.239
Planta-ego ΔRT	0.307	0.099
Planta-allo ΔRT	0.359	0.051

Ego, egocentric; allo, allocentric; RT, response time; RC, rate of change.

The bold values indicate the statistically significant difference values.

**Figure 7:**
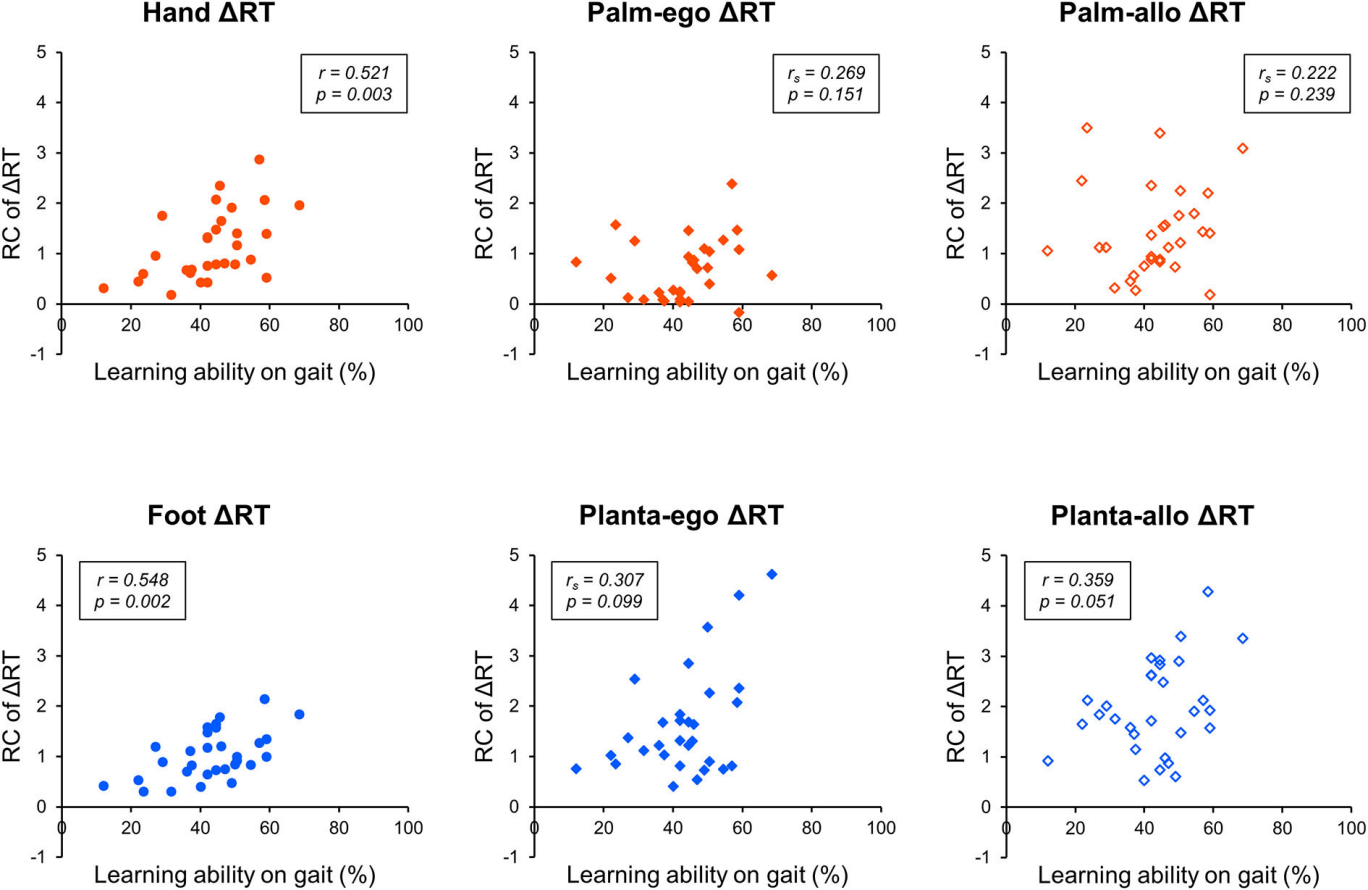
Scattergram representing the correlation between the RC of ΔRT and adaptive motor learning ability on gait. The x-axis denotes the learning ability on gait evaluated by the split-belt treadmill walking task. The y-axis denotes the rate of change (RC) of response time (ΔRT) measured by the mental rotation task.

Next, considering the RC of ΔRT, a parametric test of correlation analysis revealed statistically significant positive relationships between the adaptive learning abilities on gait and the RC of hand ΔRT (r = 0.521, *p* = 0.003) or foot ΔRT (r = 0.548, *p* = 0.002), but not the RC of planta-allo ΔRT (r = 0.359, *p* = 0.051). In addition, non-parametric correlation analysis showed that individual adaptive learning abilities during gait adaptation were not related to the RC of palm-ego ΔRT (r_s_ = 0.269, *p* = 0.151), palm-allo ΔRT (r_s_ = 0.222, *p* = 0.239), or planta-ego ΔRT (r_s_ = 0.307, *p* = 0.099; Table 2, Figure 7). These findings suggest that the RC of ΔRT only in dorsal body part images is correlated with the capability of motor adaptation on gait.

## Discussion

The main purpose of the present study was to examine the relationship between several RT values obtained from an MR task and the adaptive motor learning ability of gait, which was used to represent individual differences in the ability of motor adaptation. Irrespective of the body part visual stimulus, a positive correlation was found between the RC of ΔRT of the MR task using dorsal hand and foot images and the adaptive learning ability of gait, indicating that the slower the participants mentally transformed the body part images in a two-dimensional plane during the MR task, the worse was their ability to acquire a novel walking pattern.

A possible interpretation is that the commonality of brain regions and functions in both the MR and split-belt treadmill walking tasks contributed to this result. Neuroimaging studies using fMRI have demonstrated that the MR task of the hands is associated with activity in the prefrontal, precentral, postcentral, and parietal regions (de Lange et al., 2006; Creem-Regehr et al., 2007; Corradi-Dell'Acqua et al., 2009; Perruchoud et al., 2016; Hamada et al., 2018; Qu et al., 2018). The investigation conducted by Hinton et al. (2019) evaluated brain activation during split-belt treadmill walking using PET and reported increased activity of the supplementary motor areas (SMAs), posterior parietal cortex (PPC), anterior cingulate cortex, and anterior lateral cerebellum compared to tied-belt normal walking. These prior findings suggest that the mutually related brain regions, especially the motor-related area and parietal association area, play an essential role in the two distinct tasks.

Experiments that use non-invasive brain stimulation methods, such as transcranial magnetic stimulation (TMS) and transcranial direct current stimulation (tDCS), to examine the role of SMA and PPC during either the MR or split-belt treadmill walking task can emphasize the functional contribution of these brain regions to the two tasks. Although object stimuli were used in the MR tasks, the application of repetitive TMS (Harris and Miniussi, 2003) to disrupt the cortical activity in the right PPC or tDCS (Foroughi et al., 2014; Zhu et al., 2021) to modulate the brain region, interfered with MR task performance such as RT (Harris and Miniussi, 2003; Zhu et al., 2021) or accuracy (Foroughi et al., 2014; Zhu et al., 2021). In addition, an online TMS train (5 pulses at 10 Hz) to the SMA during the MR task was reported to improve the MR task performance (Cona et al., 2017). In a study on split-belt treadmill walking, Young et al. (2020) demonstrated that when the PPC was suppressed using tDCS prior to walking, participants required more steps to adapt to split-belt walking, indicating that the PPC plays a causal role in adaptation learning of gait. In summary, it is plausible that individual differences in the brain function of the SMA and PPC, which are commonly engaged in MR tasks and split-belt treadmill walking, contributed to the correlation of results between the two tasks.

In this study, we found that the correlation was only detected with the RC of ΔRT, which denotes the discrepancy between the RT for egocentric and allocentric views, not ΔRT itself. Prior research has suggested that the MR task involves several processes: (1) visual encoding, (2) transformation, (3) comparison, (4) decision making, and (5) motor response generation (Seurinck et al., 2004). In accordance with a previous study (Mochizuki et al., 2019; Nagashima et al., 2019), ΔRT was calculated by subtracting the RT for arrows on the same side from the RTs for right/left hand and foot images. As shown in our correlation and comparison results between the RT for arrow images and dorsal body part images of egocentric view, we can confirm that the RT for the arrow image affected the RT for the body part image, and was significantly slower than that for the body part image. These results can be interpreted that the RT for the arrow image reflected the more unmixed processes: decision making or especially motor response generation, than the RT for the body part image. Thus, the impact of the processes from decision making to motor response generation during MR tasks on individual RT values could be excluded or diminished owing to this data handling (Mochizuki et al., 2019). In addition, laterality judgment tasks differed from same-different judgment tasks (Mibu et al., 2020), which require participants to judge whether the laterality of two images displayed concurrently is the same or different. Thus, the impact of the comparison process to decide whether the two images were coincidental on the acquired RT values was also considered to be minimal in the current study. Based on these considerations, the ΔRT value obtained by our data processing appears to signify the time taken for the processes of visual encoding and transformation.

In contrast, the RC of ΔRT was processed by subtracting the ego-view ΔRT from the allo-view ΔRT and then dividing this value by the ego-view ΔRT for data analyses. Because the correlation with the individual adaptive learning ability of gait was restricted to the RC of ΔRT in the dorsal side (dorsal hand and dorsal foot) images, the comparable patterns of RT change in the dorsal side may have affected our noteworthy finding. In practice, angle rotation-dependent RT changes in the dorsal hand image were fairly congruent with those in the dorsal foot image, as shown in Figure 5. Conson et al. (2013) deemed the RT in the upright image (0°-orientation) to be involved in the perceptual (visual) encoding process; in the present study, specifically, all RTs of the dorsal hand and foot images in the egocentric view were similar. Thus, it may be suggested that in the egocentric view of the dorsal body part, a time window related to the visual encoding process was substantially occupied, and transformation or MR processing of the visual stimulus was not performed to judge the laterality of body parts. Contrarily, the RTs of dorsal side images in the allocentric view were significantly slower than those in the egocentric view, suggesting that in the allocentric view, the process of mentally rotating the displayed stimulus was used to judge the laterality of body parts.

The activity of the parieto-frontal network reportedly increases according to the biomechanical constraints of the depicted hand movements, even when the amount of stimulus rotation was equivalent (de Lange et al., 2006). Additionally, activation of the parietal and frontal cortices has also been reported to be modulated by increasing the degree of rotation in the MR task (Creem-Regehr et al., 2007). In this study, the body part images from the allocentric perspective also had the biomechanical complexity to move them internally and increase the magnitude of rotation angle, compared to those from an egocentric perspective. Taken together, the extracted RT values from our data handling (i.e., the RC of ΔRT in dorsal side images) appear to be related to the activation of the parieto-frontal circuits and strictly reflect the component of the transformation process included in the MR task, which might exclude the factors of other cognitive processes.

Although we speculated that on the ventral side, the relationship between the RC of ΔRT in the palmar and plantar images and individual adaptive learning ability could be more apparent than in dorsal side images because of its complexity in mentally transforming the body part images, the RC of ΔRT in ventral side images showed no statistically significant correlation with the adaptive motor learning ability. The behavioral research using the whole-body image by Conson et al. (2014) demonstrated that patients with Parkinson's disease were selectively impaired to judge hand laterality in the dorsal side image of human figures; however, they showed comparable patterns to healthy individuals when performing the hand laterality judgment in the ventral side image of human figures. This finding indicates that the factor of the image plane (dorsal vs. ventral side) is differentially affected by one's own body representation. In the present study, as Conson et al. (2014) speculated, because the dorsal side images can activate embodied simulation process more instinctively than the ventral side images, the RC of ΔRT in the dorsal side images alone appeared to correlate with the individual adaptive learning ability. Additionally, considering the task difficulty of the MR task, Gardony et al. (2017) attempted to scrutinize cognitive strategies in the MR task using time-frequency analysis of electroencephalography and demonstrated that the usage of cognitive strategy differed from the task difficulty of the MR task. In fact, both the ego-view and allo-view ΔRTs in ventral side images were slower than the ego-view ΔRTs in dorsal side images, indicating that the MR task using ventral side images is more difficult to judge the body part laterality, than using dorsal side images. Because different cognitive strategies in motor simulation, such as visual working memory (Gardony et al., 2017), or more composite processes, such as the three-dimensional transformation process, were presumably required to complete the MR task using ventral side images relative to the dorsal side, it is assumed that the RC of ΔRT in the ventral side images was not involved in adaptive motor learning capability on gait, and did not directly reflect the same.

Regarding the RC of ΔRT in ventral side images, a curious finding was also detected. Although the correlation results did not reach the significance level, the RC of ΔRT in only the foot, and not the hand images, tended to be associated with the motor adaptation ability on gait (*p* < 0.1). The split-belt treadmill walking task is a whole-body movement that primarily requires motor adaptations in the lower extremities. Given the absolute impact of physical function/ability on motor learning in general, individual differences in lower limb function/ability, rather than upper limb function, may have affected the different correlation results between the upper and lower limbs in this study. Physical function-dependent performance of the MR task has been well investigated (Fiorio et al., 2006; Katschnig et al., 2010; Ionta et al., 2016; Scandola et al., 2019), which may partially support the results of our study.

The present study has some limitations. We used locomotor adaptation learning as a specific instance of motor learning. To generalize our results to other forms of motor learning, future studies should clarify the direct association between the MR task using corporal images and implicit/explicit motor adaptation tasks with upper/lower extremities. Besides, we tried to clarify what cognitive process or brain function was reflected in the RTs obtained from the MR task, especially in relation to the ability of motor adaptation. Although it was found from the behavioral data that the process or ability to transform corporal visual stimuli was a crucial element related to actual motor adaptation ability, the brain regions or networks precisely associated with this transformation process were not clarified in this experiment. Therefore, further research is required to clarify this point in consideration of intra- and inter-participant data variability. This would provide critical information in order to develop adaptive learning ability or promote the learning process of gait using the MR task with body part images.

In conclusion, the RC of ΔRT in the dorsal, but not ventral, side images, which is assumed to reflect the genuine transformation process of the MR task, correlated with the actual motor adaptation ability alone. In contrast, there was no significant relationship between the ΔRT of the MR task and adaptive learning ability of gait, irrespective of body parts or rotation angles (perspectives). This finding indicates that, while the types or angles of displayed pictures are not essential elements in the MR task relevant to individual adaptive learning capability, individual differences in the processing ability to transform spatial sensory (visual) information is a crucial element related to adaptive learning ability. Our novel findings advance the current understanding of the elements included in the MR task and its relationship to motor adaptation/learning ability.

## Data availability statement

The datasets generated and analyzed during the current study are available from the corresponding author upon reasonable request.

## Ethics statement

The studies involving human participants were reviewed and approved by the Ethics Committee of the Kawasaki University of Medical Welfare. The participants provided their written informed consent to participate in this study.

## Author contributions

TI and MK designed the experiments. TI performed data collection and drafted the manuscript. TI, MK, and TH were involved in the data analysis. TI, MK, TH, DK, and AT were involved in the conception of this study. All authors interpreted the data, critically revised the manuscript, and approved the final version of the manuscript for submission.

## Funding

This work was supported by JSPS KAKENHI (Grant Number: JP18K17780 to TI) and the Kawasaki University of Medical Welfare Scientific Research Fund.

## Conflict of interest

The authors declare that the research was conducted in the absence of any commercial or financial relationships that could be construed as a potential conflict of interest.

## Publisher's note

All claims expressed in this article are solely those of the authors and do not necessarily represent those of their affiliated organizations, or those of the publisher, the editors and the reviewers. Any product that may be evaluated in this article, or claim that may be made by its manufacturer, is not guaranteed or endorsed by the publisher.
